# Supplementary material for: This is the way the world ends; not with a bang but a whimper: Estimating the number and ongoing rate of extinctions of Australian non-marine invertebrates
Source: Camb Prism Extinct. 2024 Dec 9;2:e23. doi: 10.1017/ext.2024.26 (PMC11895748; doi:10.1017/ext.2024.26)
Supplement: Woinarski et al. supplementary material [file S2755095824000263sup001.zip › Woinarski_etal_Supplementary.docx]

***This is the way the world ends; not with a bang but a whimper*: estimating the number and ongoing rate of extinctions of Australian non-marine invertebrates**

**Appendix S1. Supplementary material.**

Note that all calculations, including Monte-Carlo simulations, were conducted with functions in Excel.

**(1) Number of Australian endemic terrestrial and freshwater invertebrate species**

We derived five estimates of the number of Australian endemic invertebrate species, using values from the published literature (Table 1). From the five derived estimates, we took the mean (595,359) to approximate the most-likely true value and used the range of the values (172,259–1,822,327) as the plausible bounds, which we interpret as equivalent to 95% confidence limits.

We created a simulated distribution representing the possible values of the number of Australian endemic invertebrate species. The distribution needed to be strongly right-skewed, with a median matching the most-likely value (595,359), and 2.5% and 97.5% quantiles matching the lower and upper plausible bounds (172,259 and 1,822,327), respectively. We sampled a normal distribution, with mean of 13.381 and standard deviation of 0.5422, and then applied the following transformation to each sample: *y* = *e^x^* – 51,477. The resulting distribution (*n* = 100,000) is shown in Figure S1.

Figure S1. Simulated distribution (*n* = 100,000) of number of Australian endemic invertebrate species, based on the most-likely value of 595,359 (empirical median is 596,081), and plausible bounds of 172,259─1,822,327 (empirical 2.5% and 97.5% quantiles are 172,401 and 1,826,205, respectively).

**(2) Proportional extinction rates: derived from Australian extinction rates for other taxonomic groups**

We took the number of extinct species in taxonomic groups other than invertebrates from Woinarski et al. (2019), but this is conservative as the tally given there is the number of formally recognised extinctions (see Figure 1). For ***plants***, we then reduced the tally given in that source by three species (from 38 to 35) on the basis of the recent re-discovery of two species (e.g., Forster 2019) and taxonomic review that has led to one extinct species being lumped with an extant species (Ayre et al. 2021): here, we use 35 species as the minimum and plausible number of plant extinctions and, recognising many other plant species have been long unrecorded but not yet recognised as extinct (Silcock et al. 2020), 40 species as an upper limit. The number of endemic plant species (18,714) is taken from Gallagher et al. (2023). Hence the extinction rate for Australian plants is estimated to be between from 0.19% to 0.21%. For ***freshwater fish***, Woinarski et al. (2019) listed no extinctions, but one species as extinct in the wild. This is likely to be an under-estimate given a spate of recent taxonomic reviews that have split many species into series of short-range endemics, the marked susceptibility of many native fish to impacts of introduced fish species, and severe degradation of many aquatic environments. Here, we use zero species as the minimum and one species as the plausible number of fish extinctions, and – recognising likely extinctions not yet documented – two species as the upper limit (with the additional species being the informally recognised extinction of the undescribed Kangaroo River Macquarie perch: M. Lintermans *pers. comm.*). The number of endemic freshwater fish species (315) is taken from Lintermans et al. (2020); hence the extinction rate for endemic freshwater fish is 0.32% (range 0 to 0.64%). For ***frogs***, Gillespie et al. (2020) documented extinction of five species, and a tally of 243 species in Australia (i.e., 2.06% extinctions). Woinarski et al. (2019) listed one ***reptile*** extinction and two species as extinct in the wild. Several other species have not been recorded for at least 50 years so may be unrecognised extinctions (Chapple et al. 2019; Garnett et al. 2022). Here, we use one species as the minimum and three species as the plausible number of reptile extinctions, and – recognising likely extinctions not yet documented – five species as the upper limit. There is a high rate of descriptions of new Australian reptile species (Chapple et al. 2019), so the actual number of endemic reptiles is poorly resolved. Chapman (2009) estimated 1,100 reptile species present in Australia, with 92% of these being endemic; so, we assume 1,012 endemic reptile species. Hence the estimated proportion of reptile extinctions is 0.30% (range 0.10% to 0.49%). Woinarski et al. (2024a) documented nine ***bird*** extinctions in Australia from a tally of 363 endemic species, although recent taxonomic revision inferred that two of these species may be relegated to subspecies (Cibois et al. 2020). Here, we recognise seven species as the minimum number of bird extinctions, and nine as the plausible and higher bound. Hence, we estimate the proportional extinction in Australian endemic birds is 2.48% (range 1.93% to 2.48%). For ***mammals***, Woinarski et al. (2019) listed 34 extinctions, but subsequently one of these has been lumped with an extant species (Roycroft et al. 2021), and taxonomic review has described three new species, all now extinct (Newman-Martin et al. 2023). A recent assessment concluded that an additional species, the Christmas Island shrew *Crocidura trichura* is also highly likely to be extinct (Woinarski et al. 2024b). Here, we recognise 36 as the minimum and plausible, and 37 as the upper bound for the number of mammal extinctions (Burbidge 2024). The number of Australian endemic mammal species is 291; hence the proportional extinction rate for mammals is 12.37% (range 12.37% to 12.72%).

For all taxonomic groups, the number of known extinctions and number of known species are subject to change as a result of new discoveries and taxonomic review. This taxonomic refinement has included some recent recognition of dark extinctions in Australian mammals (resulting in increase in the known extinction rate for mammals), demonstrated in part because of subfossils (Newman-Martin et al. 2023). Comparable subfossil evidence is far less likely to be available for dark extinctions in plant, fish, frog and reptile species, so it is highly likely that the extinction rates for these groups are under-estimates of actual extinctions.

Based on these assessments, the plausible ranges of proportional extinction rates for Australian endemic species in other taxonomic groups (see main text) are summarised in Table S1.

Table S1. Summary of plausible extinction rates for Australian endemic species in other taxonomic groups.

| Taxonomic group | Lower plausible % EX | Best guess % EX | Higher plausible % EX |
| --- | --- | --- | --- |
| Plants | 0.19 | 0.19 | 0.21 |
| Freshwater fish | 0 | 0.31 | 0.64 |
| Frogs | 2.06 | 2.06 | 2.06 |
| Reptiles | 0.1 | 0.3 | 0.49 |
| Birds | 1.93 | 2.48 | 2.48 |
| Mammals | 12.4 | 12.4 | 12.7 |
| Averaged across groups | [excludes atypical rate for mammals]  0.85 | [excludes atypical rate for mammals]  1.07 | 3.10 |

We created a simulated distribution representing the possible values of proportional extinction rate of Australian endemic species across these other taxonomic groups. The distribution needed to be strongly right-skewed, with a median matching the most-likely value (1.07%), and 2.5% and 97.5% quantiles matching the lower and upper plausible bounds (0.85% and 3.10%), respectively. We sampled a normal distribution, with mean of -6.0456 and standard deviation of 1.1645, and then applied the following transformation to each sample: *y* = *e^x^* + 0.0083. The resulting distribution (*n* = 100,000) is shown in Figure S2.

Figure S2. Simulated distribution (*n* = 100,000) of proportional extinction of Australian endemic species across other taxonomic groups, based on the most-likely value of 1.07% (empirical median is 1.07%), and plausible bounds of 0.85─3.10% (empirical 2.5% and 97.5% quantiles are 0.85% and 3.16%, respectively).

**(3) Proportional extinction rates: derived from global invertebrate extinction rates**

We created a simulated distribution representing the possible values of proportional extinction rate of the world's invertebrates. Our assumed most-likely value was simply the mid-point of the lower and upper plausible bounds, which meant that the distribution was symmetrical; hence we used a normal distribution, without any need for a transformation to induce skew. The normal distribution had a mean matching our best-guess (i.e. 1.99%) and standard deviation (SD) of:

SD = 0.5 × (*upper plausible bound* – *lower plausible bound*) / 1.96.

The resulting distribution (*n* = 100,000) is shown in Figure S3.

Figure S3. Simulated distribution (*n* = 100,000) of proportional extinction rates for global invertebrates, based on the most-likely value of 1.99% (empirical median is 1.99%), and plausible bounds of 1.40─2.57% (empirical 2.5% and 97.5% quantiles are 1.40% and 2.58%, respectively).

**(4) Uncertainty in the number of extinctions of Australian endemic invertebrate species**

We used a Monte Carlo simulation approach to characterise the overall uncertainty in the total number of Australian endemic invertebrate species extinct (1788─2024). Our analysis of uncertainty was restricted to that derived from uncertainty in two key parameters: (1) the number of Australian endemic terrestrial and freshwater invertebrate species extant in 1788; and (2) the proportion of those species that have become extinct since 1788. The simulated distributions of these parameters are described in the preceding sections (and shown in Figures S1–S3). Given that the proportion of species extinct was estimated using two different approaches (Approach 1: using the Australian extinction rate for non-invertebrates; and Approach 2: using the global extinction rate for invertebrates), we used a separate distribution for each (Figures S2–S3).

We randomly sampled from the three distributions n=100,000 times, allowing us to generate 100,000 estimates of the total number of extinct Australian endemic invertebrate species using Approach 1, and 100,000 estimates using Approach 2 (Figure S4). For each random realisation of the simulated distributions, we recalculated the total number of extinct Australian endemic invertebrate species, and we report the 2.5% and 97.5% quantiles. We report these separately for Approaches 1 and 2 and assume that the lesser of the two 2.5% quantile estimates is the lower plausible bound of the total number of extinct Australian endemic invertebrate species, and that the greater of the two 97.5% quantile estimates is the upper plausible bound.

1. Approach 1: Proportional extinction rate inferred from Australian extinction rate for non-invertebrates

1. Approach 2: Proportional extinction rate inferred from global extinction rate for invertebrates

Figure S4. The distribution of estimates of the total number of extinct Australian endemic invertebrate species, derived using Monte Carlo simulation. Two separate approaches were used to estimate the proportion of species extinct: (a) Approach 1: using the Australian extinction rate for non-invertebrates; and (b) Approach 2: using the global extinction rate for invertebrates), and the resulting distribution is shown separately for each.

**(5) Estimating annual rate of extinctions**

Our default setting is that extinction rate is constant over time. However, more plausibly, the rate of extinctions in any area is likely to be driven by changes over time in the extent and intensity of threats operating in that area. We think it is impossible to detail and quantify such changes in threat load over time across an area as large and diverse as Australia. As a simple, single potential surrogate for changes over time in the overall threat load, we use growth in Australia’s human population size over time, and mirror that curve to estimate the number of extinctions in a given year as a percentage of all extinctions over the period 1788-2024 (Figure S5).

Figure S5. Estimated change in human population size in Australia since 1800 (left axis), and the estimated proportion of modern extinctions (1788–2024) that have occurred in each year (right axis), assuming that the annual extinction rate is proportional to human population size. The source of the population data is: <https://www.statista.com/statistics/1066666/population-australia-since-1800/>.

**References**

Ayre BM, Hayashi T, Phillips RD and Reiter N (2021) The Kiandra leek orchid is the previously presumed extinct mignonette leek orchid (Orchidaceae; Orchidoideae): evidence from morphological comparisons. *Phytotaxa* 528, 71-83.

Burbidge AA (2024) Australian terrestrial mammals: how many modern extinctions? *Astralian Mammalogy* 46, AM23037.

Chapman AD (2009) Numbers of living species in Australia and the world. Canberra: Australian Biological Resources Study.

Chapple DG, Tingley R, Mitchell NJ, Macdonald SL, Keogh JS, Shea GM, Bowles P, Cox NA and Woinarski JCZ (2019) *The Action Plan for Australian Lizards and Snakes 2017*. Melbourne: CSIRO Publishing.

Cibois A, Vallotton L, Ericson PGP, Blom MPK and Irestedt M (2020) Genetic and radiographic insights into the only known mounted specimen of Kangaroo Island Emu. *Revue suisse de Zoologie* 126, 209-217.

Forster PI (2019) Rediscovery of the previously Extinct *Marsdenia araujacea* F.Muell. (Apocynaceae). *Austrobaileya* 10, 539-540.

Gallagher RV, Allen SP, Govaerts R, Rivers MC, Allen AP, Keith DA, Merow C, Maitner B, Butt N, Auld TD, Enquist BJ, Eiserhardt WL, Wright IJ, Mifsud JCO, Espinosa-Ruiz S, Possigham H and Adams VM (2023) Global shortfalls in threat assessments for endemic flora by country. *Plants, People, Planet* 5, 885-898.

Garnett ST, Hayward-Brown BK, Kopf RK, Woinarski JCZ, Cameron KA, Chapple DG, Copley P, Fisher A, Gillespie G, Latch P, Legge S, Lintermans M, Moorrees A, Page M, Renwick J, Birrell J, Kelly D and Geyle HM (2022) Australia’s most imperilled vertebrates. *Biological Conservation* 270, 109561.

Gillespie GR, Roberts JD, Hunter D, Hoskin CJ, Alford RA, Heard GW, Hines H, Lemckert F, Newell D and Scheele BC (2020) Status and priority conservation actions for Australian frog species. *Biological Conservation* 247, 108543.

Lintermans M, Geyle HM, Beatty S, Brown C, Ebner B, Freeman R, Hammer M, Humphreys WF, Kennard MJ, Kern P, Martin K, Morgan D, Raadik TA, Unmack PJ, Wager R, Woinarski JCZ and Garnett ST (2020) Big trouble for little fish: identifying Australian freshwater fishes in imminent risk of extinction. *Pacific Conservation Biology* 26, 365-377.

Newman-Martin J, Travouillon KJ, Warburton N, Barham M and Blyth AJ (2023) Taxonomic review of the genus *Dasycercus* (Dasyuromorphia: Dasyuridae) using modern and subfossil material; and the description of three new species. *Alcheringa: An Australasian Journal of Palaeontology* 47, 624-661.

Roycroft E, MacDonald AJ, Moritz C, Moussalli A, Miguez RP and Rowe KC (2021) Museum genomics reveals the rapid decline and extinction of Australian rodents since European settlement. *Proceedings of the National Academy of Sciences* 118(27), e2021390118.

Silcock JL, Field AR, Walsh NG and Fensham RJ (2020) To name those lost: assessing extinction likelihood in the Australian vascular flora. *Oryx* 54, 167-177.

Woinarski JCZ, Braby MF, Burbidge AA, Coates D, Garnett ST, Fensham RJ, Legge SM, McKenzie NL, Silcock JL and Murphy BP (2019) Reading the black book: the number, timing, distribution and causes of listed extinctions in Australia. *Biological Conservation* 239, 108261.

Woinarski JCZ, Legge SM and Garnett ST (2024a) Extinct Australian birds: numbers, characteristics, lessons and prospects. *Emu - Austral Ornithology* 124, 8-20.

Woinarski JCZ, Tiernan B and Legge SM (2024b) Should the Christmas Island shrew *Crocidura trichura* be considered extinct? *Australian Mammalogy* 46, AM23033.
