# Supplementary figures and images for: This is the way the world ends; not with a bang but a whimper: Estimating the number and ongoing rate of extinctions of Australian non-marine invertebrates
Source: Camb Prism Extinct. 2024 Dec 9;2:e23. doi: 10.1017/ext.2024.26 (PMC11895748; doi:10.1017/ext.2024.26)

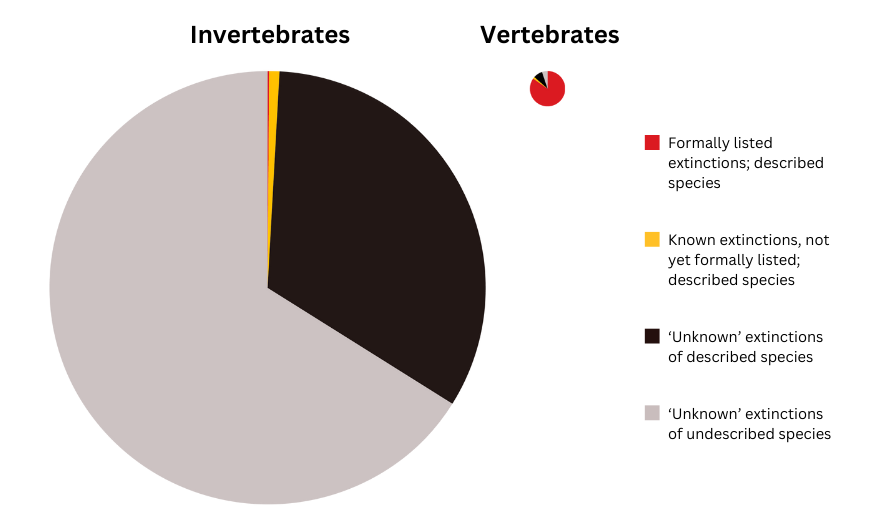

Supplement: Woinarski et al. supplementary material [file S2755095824000263sup001.zip › Figure 1_v2.png]
